# Sex differences in multisensory speech processing in both typically developing children and those on the autism spectrum

**DOI:** 10.3389/fnins.2015.00185

**Published:** 2015-05-27

**Authors:** Lars A. Ross, Victor A. Del Bene, Sophie Molholm, Hans-Peter Frey, John J. Foxe

**Affiliations:** ^1^The Sheryl and Daniel R. Tishman Cognitive Neurophysiology Laboratory, Department of Pediatrics, Children's Evaluation and Rehabilitation Center, Albert Einstein College of Medicine & Montefiore Medical CenterBronx, NY, USA; ^2^The Gordon F. Derner Institute of Advanced Psychological Studies, Adelphi UniversityGarden City, NY, USA; ^3^Ferkauf Graduate School of Psychology, Albert Einstein College of MedicineBronx, NY, USA; ^4^The Dominick P. Purpura Department of Neuroscience, Rose F. Kennedy Intellectual and Developmental Disabilities Research Center, Albert Einstein College of MedicineBronx, NY, USA; ^5^Division of Neurocritical Care, Department of Neurology, Columbia University Medical CenterNew York, NY, USA

**Keywords:** autism spectrum disorder, cross-modal, development, gender, audio-visual, ASD, language, speech perception

## Abstract

**Background:** Previous work has revealed sizeable deficits in the abilities of children with an autism spectrum disorder (ASD) to integrate auditory and visual speech signals, with clear implications for social communication in this population. There is a strong male preponderance in ASD, with approximately four affected males for every female. The presence of sex differences in ASD symptoms suggests a sexual dimorphism in the ASD phenotype, and raises the question of whether this dimorphism extends to ASD traits in the neurotypical population. Here, we investigated possible sexual dimorphism in multisensory speech integration in both ASD and neurotypical individuals.

**Methods:** We assessed whether males and females differed in their ability to benefit from visual speech when target words were presented under varying levels of signal-to-noise, in samples of neurotypical children and adults, and in children diagnosed with an ASD.

**Results:** In typically developing (TD) children and children with ASD, females (*n* = 47 and *n* = 15, respectively) were significantly superior in their ability to recognize words under audiovisual listening conditions compared to males (*n* = 55 and *n* = 58, respectively). This sex difference was absent in our sample of neurotypical adults (*n* = 28 females; *n* = 28 males).

**Conclusions:** We propose that the development of audiovisual integration is delayed in male relative to female children, a delay that is also observed in ASD. In neurotypicals, these sex differences disappear in early adulthood when females approach their performance maximum and males “catch up.” Our findings underline the importance of considering sex differences in the search for autism endophenotypes and strongly encourage increased efforts to study the underrepresented population of females within ASD.

## Introduction

Autism Spectrum Disorders (ASDs) are diagnosed in considerably greater numbers of males than females, with estimated ratios in the range of four affected males for every female (CDC, 2014). The mechanisms that give rise to this male bias are not well understood and are the subject of much current debate (e.g., Baron-Cohen et al., 2009; Fombonne, 2009; Werling and Geschwind, 2013). Several biological and non-biological theories have been proposed.

Non-biological models attribute differences in prevalence rate to biases introduced by differences in the presentation of ASD symptoms. Males with ASD have been reported to show more “externalizing behavior” including hyperactivity, aggressive behaviors, and repetitive and stereotyped behaviors and interests (Giarelli et al., 2010; Bölte et al., 2011; Hattier et al., 2011; Mandy et al., 2012; Solomon et al., 2012; Szatmari et al., 2012). On the other hand, females diagnosed with ASD present with more “internalizing behaviors” such as anxiety and depression (Hattier et al., 2011; Solomon et al., 2012). It therefore seems quite likely that the more socially disruptive behaviors in males have a higher likelihood to motivate parents or caretakers to seek clinical evaluations. In females, ASD symptoms are diagnosed when associated with more severe intellectual disabilities. In addition, high functioning ASD in females may be masked by their higher social abilities causing them to remain undiagnosed (Russell et al., 2011; Dworzynski et al., 2012).

Several biological models have been proposed to explain sex differences in ASD prevalence. The Extreme Male Brain (EMB) theory (e.g., Baron-Cohen, 2002) proposes that factors inherent in the male genotype and development that give rise to typically observed sexual dimorphisms in cognition (e.g., empathy and systemizing; Asperger and Frith, 1991) may be exaggerated in people affected with ASD giving rise to disordered social behavior (Baron-Cohen et al., 2003, 2005; Baron-Cohen and Wheelwright, 2004). This proposed “masculinization” can be observed in overt behavior (Ingudomnukul et al., 2007; Knickmeyer et al., 2008) and at the levels of brain structure and function (Lai et al., 2013) and may be linked to the expression of sex-hormones (Auyeung et al., 2006, 2009). Another model proposes that females with ASD carry a higher mutational load than affected males, but that the minimum liability threshold sufficient to cause ASD is higher in females. Evidence in support of this model has been mixed (Goin-Kochel et al., 2007; Hallmayer et al., 2011), but recently, some fairly convincing support emerged from a study by Robinson et al. (2013). The central premise of the Robinson study was that if a female “protective” effect exists, then a simple prediction would be that siblings of females with very high autistic trait scores (i.e., those above the 90th percentile) should show greater autistic trait scores than siblings of males with similarly high trait scores. This is exactly what was found in this large study of almost 10,000 twin pairs. There is also support for this protective notion from studies in females with Turner syndrome, a chromosomal abnormality where one of the X chromosomes is either missing or partially deleted (Bondy et al., 2012). Skuse and colleagues showed that social difficulties in this syndrome were predicated upon the parent of origin of the functioning X chromosome (Skuse et al., 1997). That is, those girls with a paternally derived X showed better social abilities than those with a maternally derived X, pointing to an imprinted genetic locus (or loci) for social cognitive functions expressed only on the paternal X. Of course, all males inherit their X chromosome from the mother, and so they won't express this socially protective gene (or genes), and their threshold for ASD would therefore be lower than it is in females (Skuse, 2000).

The study of sex differences in ASD traits is challenged by the relatively lower prevalence of females diagnosed with ASD and complicated by developmental changes and biases inherent in the diagnostic process due to differences in the presentation of symptoms. These circumstances may have led to inconsistencies in reports on sex differences in core ASD traits with some studies reporting evidence for sex differences in core diagnostic features (McLennan et al., 1993; Carter et al., 2007; Hartley and Sikora, 2009; Lai et al., 2011; Mandy et al., 2012) and other studies providing evidence to the contrary (Tsai and Beisler, 1983; Pilowsky et al., 1998; Holtmann et al., 2007; Solomon et al., 2012). Biological and non-biological models of sex differences in ASD are clearly not mutually exclusive, and both have important implications for the investigation of genetics, brain function and their relationship to the overt symptoms of ASD. These implications extend to research on ASD traits in the neurotypical population as there is mounting evidence for the heritability of ASD traits in unaffected individuals (Robinson et al., 2011; Lundström et al., 2012) and it has been suggested that “*at least some of the genetic and environmental factors associated with ASD are the same as those that cause individual differences in autism-like behavior below the clinical threshold*.” (Robinson et al., 2013). If indeed autistic traits are represented on a continuous spectrum that extends into the unaffected population then the sexual dimorphism in ASD characteristics should as well. In other words, if there is a sexual dimorphism of core ASD symptoms in individuals with an ASD diagnosis, then these sex differences would likely also manifest as different distributions of ASD traits in the unaffected population. For example, Matsuyoshi et al. (2014) examined sensitivity to eye gaze direction in 128 unaffected adults (64 females), a task in which individuals with ASD display robust deficits (see Senju and Johnson, 2009 for a review). The investigators found that individual differences in autistic traits predicted performance in this task in male but not female participants suggesting that direct-gaze perception may not constitute an autistic endophenotype in both sexes. Lai et al. (2012) studied four key cognitive domains including mentalizing and emotion perception, executive function, perceptual attention to detail and motor function in 128 male and female adults with and without ASD (32 per group). They found that deficits in mentalizing and facial emotion perception in individuals with ASD compared to controls were similar in both sexes. However, attention to detail and dexterity involving executive function were found to be impaired only in male ASD participants. The authors suggested that performance in the social cognitive domain is equally impaired in male and female individuals with ASD, whereas sex differences are observed in non-social cognitive domains. These findings lend support to the notion of sex differences in the disease phenotype and associated traits in the “normal” population and represent compelling reasons to consider sex differences when studying ASD traits in affected and unaffected samples.

In a previous cross-sectional study of 84 children with ASD and 142 neurotypical children between the ages of 5 and 17, we provided strong support for severe multisensory deficits in audiovisual speech perception during childhood and a subsequent recovery around 12 years of age in children with an ASD (Foxe et al., 2015). These large and consistent deficits in audiovisual gain between 5 and 12 years of age could not be explained by unisensory speech perception deficits alone or differences in eye-gaze as assessed with eye-tracking. These findings raise important questions about the neural mechanisms underlying these observed deficits, their possible heritability and link to biological sex. An advanced understanding of sex differences in ASD may benefit our understanding of the genetic, neurobiological and environmental factors involved in the development of ASD. Basic research on sex differences in ASD has practical implications for therapeutic intervention and may inform clinicians to delineate more personalized treatments for this diverse disorder.

In the current study we assessed sex differences in the perception of auditory, visual and audiovisual speech presented in varying levels of noise in typically developing children between the ages of 5 and 17 years of age. We also explored possible sex differences in speech perception in neurotypical adults. We finally examined sex differences in speech perception performance in a sample of ASD children between 8 and 17 years of age.

## Methods

### Participants

Our first analysis involved 102 typically developing children in the age range from 5 to 17 years of age. The 55 male participants in our sample had a mean age of *M*_age_ = 12.02 years (*SD*_age_ = 3.2) and a mean full IQ (FIQ) of *M*_FIQ_ = 112.02 (*SD*_FIQ_ = 12.01). The 47 females were on average *M*_age_ = 11.36 (*SD*_age_ = 3.55) years of age with a mean FIQ of *M*_FIQ_ = 107.19 (*SD*_FIQ_ = 15.95). The data of these participants represent a subset of a larger sample and were selected because these participants were assessed with the Wechsler Abbreviated Scales of Intelligence (WASI) and therefore allowed the inclusion of FIQ as a covariate. An additional analysis was carried out in a sample of 28 male (*M*_age_ = 26.16; *SD*_age_ = 4.31) and 28 female (*M*_age_ = 25.34; *SD*_age_ = 4.23) neurotypical adults between 20 and 39 years of age. Finally, we analyzed sex differences in a sample of 58 male (*M*_age_ = 10.79; *SD*_age_ = 2.15) and 15 female (*M*_age_ = 11.87; *SD*_age_ = 2.36) children between 8 and 15 years of age who had previously been diagnosed with ASD. It should be noted that the samples of TD and ASD children reported here overlap with the samples reported in Foxe et al. (2015). A breakdown of the demographics of each of the subgroups is presented in Table 1.

**Table 1:** **Sample demographics**.

**Males**						**Females**				
	**n**	**AGE**	**VIQ**	**PIQ**	**FIQ**	**n**	**AGE**	**VIQ**	**PIQ**	**FIQ**
TDCH	55	12 (3.2)	113.4 (12.8)	107.5 (13)	112 (11.9)	47	11.4 (3.6)	109.7 (16.4)	103.9 (14.5)	107.2 (15.5)
TDAD	28	26.2 (4.31)	–	–	–	28	25.3 (4.2)	–	–	–
ASDCH	58	10.8 (2.2)	98.5 (20.6)	106.2 (17.6)	102.4 (19.2)	15	11.9 (2.4)	103.3 (16.1)	104.9 (13.5)	104.4 (13.6)

*TDCH, TD children; TDAD, TD adults; ASDCH, ASD children; n, sample size. Scores in cells represent means and standard deviations. IQ- scores were obtained in 48 male and 10 female participants with ASD. No IQ- information was obtained from adults*.

**Table 2:** **Auditory- alone performance as a function of Sex, Age, FIQ, and SNR in TD children (5–17)**.

**Source**	***SS***	***df***	***MS***	***F***	***p***	**η^2^_*p*_**
**TESTS OF BETWEEN-SUBJECTS EFFECTS**						
Age	4023.306	1	4023.306	23.174	0.000	0.191
FIQ	1354.593	1	1354.593	7.802	0.006	0.074
Sex	654.207	1	654.207	3.768	0.055	0.037
Error	17014.012	98	173.612			
**TESTS OF WITHIN-SUBJECTS EFFECTS**						
SNR	270.603	3.007	89.998	0.881	0.452	0.009
SNR × Age	2667.699	3.007	887.233	8.683	0.000	0.081
SNR × FIQ	970.518	3.007	322.778	3.159	0.025	0.031
SNR × Sex	438.081	3.007	145.699	1.426	0.235	0.014
Error	30110.154	294.66	102.185			

All participants were native English speakers. Participants were excluded from this study if they had a history of seizures or had uncorrected vision problems. TD children were excluded if they had a history of psychiatric, educational, attentional or other developmental difficulties as assessed by a history questionnaire and were also excluded if their parents endorsed six or more items of inattention or hyperactivity on a DSM-IV checklist for attention deficit disorder (with and without hyperactivity). Diagnoses of ASD were obtained by a trained clinical psychologist using the Autism Diagnostic Interview-R (ADI-R; Lord et al., 1994) and the Autism Diagnostic Observation Schedule (ADOS-G; Lord et al., 2000). All children had normal or corrected-to normal vision and audiometric threshold evaluation confirmed that all children had within-normal-limits hearing. The parents of all child participants provided written informed consent in accordance with the tenets of the 1964 Declaration of Helsinki. All procedures were approved by the institutional review board(s) of the City College of New York and the Albert Einstein College of Medicine.

### Stimuli and task

Stimulus materials consisted of digital recordings of 300 simple monosyllabic words spoken by a female speaker. This set of words was a subset of the stimulus material created for a previous experiment in our laboratory (Ross et al., 2007a) and used in previous studies (Ross et al., 2011; Foxe et al., 2015). These words were taken from the “MRC Psycholinguistic Database” (Coltheart, 1981) and were selected from a well-characterized normed set based on their written-word frequency (Kucera and Francis, 1967). The subset of words for the present experiment is a selection of simple, high-frequency words from a child's everyday environment and is likely to be in the lexicon of children in the age-range of our sample. The recorded movies were digitally re-mastered so that the length of the movie (1.3 s) and the onset of the acoustic signal were similar across all words. Average voice onset occurred at 520 ms after movie onset (*SD* = 30 ms). The words were presented at approximately 50 dBA FSPL, at seven levels of intelligibility including a condition with no noise (NN) and six conditions with added pink noise at 53, 56, 59, 62, 65, and 65 dB SPL. Noise onset was synchronized with movie onset. The signal-to-noise ratios (SNRs) were therefore NN, −3, −6, −9, −12, −15, −18 dB. These SNRs were chosen to cover a performance range in the auditory-alone condition from 0% recognized words at the lowest SNR to almost perfect recognition performance with no noise. The movies were presented on a monitor (NEC Multisync FE 2111SB) at 80 cm distance from the eyes of the participants. The face of the speaker extended approximately 6.44° of visual angle horizontally and 8.58° vertically (hairline to chin). The words and pink noise were presented over headphones (Sennheiser, model HD 555).

The main experiment consisted of three randomly intermixed conditions: In the auditory-alone condition (A) the auditory words were presented in conjunction with a still image of the speakers face; in the audiovisual condition (AV) the auditory words were presented in conjunction with the corresponding video of the speaker articulating the words. Finally, in the visual alone condition (V) only the video of the speaker's articulations was presented. The word stimuli were presented in a fixed order and the condition (the noise level and whether it was presented as A, V, or AV) was assigned to each word randomly. Stimuli were presented in 15 blocks of 20 words with a total of 300 stimulus presentations. There were 140 stimuli for the A and AV conditions respectively (20 stimuli per condition and intelligibility level) and 20 stimuli for the V condition that was presented without noise.

Participants were instructed to watch the screen and report which word they heard (or saw in the V-alone condition). If a word was not clearly understood, participants were encouraged to make their best guess. An experimenter, seated approximately 1 m distance from the participant at a 90° angle to the participant-screen axis, monitored participant's adherence to maintaining fixation on the screen. Only responses that exactly matched the presented word were considered correct. Any other response was recorded as incorrect.

### Eye tracking

Eye movements were recorded using an EyeLink 1000 system (SR Research, Ontario, Canada), at a sampling rate of 500 Hz. As described previously (Foxe et al., 2015), a small target sticker was placed on the participants' forehead, allowing the system to compensate for head movements of up to 20 cm. In order to prevent larger head movements the children had to place their heads on a comfortable chin rest. The eye tracking system was calibrated using a nine-point calibration before each set of 5 blocks of stimuli (or more often if necessary). Using the default settings, saccades and fixations were defined by the EyeLink system. Data were collected for 90 (59 male, 41 female) typically developing and 68 (58 male, 10 female) ASD participants. In the typically developing group, three datasets had to be removed (all female), while for ASD 6 datasets had to be removed (3 male, 3 female).

Custom Matlab scripts (Mathworks, Natick, MA USA) were used to analyze the Eye-tracking data. We determined the proportion of fixations on the different parts of the speaker's face. This was accomplished by selecting three rectangular patches, covering the whole face, only the mouth, or only the eyes and determining the proportion of fixations within these patches. Since lips and jaw move during speech production, the mouth region was defined vertically from the bottom of the lower jaw to just below the nose (the nasolabial angle of the philtrim). These measures were taken from the still image of the speaker before articulation started (i.e., with the mouth closed). The proportion of fixations in the different groups was statistically compared using a z-statistic for proportions (two-tailed), while numbers of fixations were compared using a *t*-test for independent samples (two-tailed). Data were analyzed for all conditions combined.

In addition to the statistical analyses, fixation distribution maps were in accordance with previous studies (e.g., Frey et al., 2011). These maps provide information about how consistent fixation locations are between participants and throughout the different trials. A value of 1 indicates that all participants fixated the same location during each fixation. Lower values indicate that participants either are not consistent in where they look or fixate different parts of the face in successive fixations. For example, if there are two small objects of interest in a scene, which are consistently fixated (equally often) by all participants, then the fixation map will have two peaks with a height of about 0.5.

### Analyses of task performance

**Figure 1:**
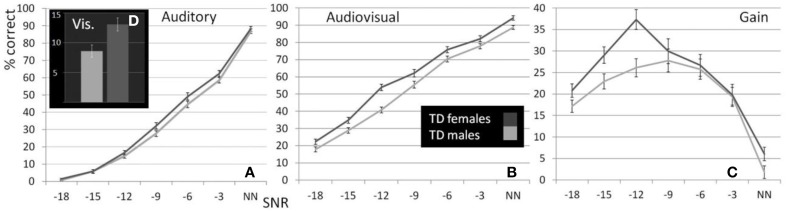
**Performance of male and female TD children**. Estimated Marginal Means (at Age 11.72 and FIQ: 109.79) for TD males and females (ages 5–17) for auditory **(A)**, visual **(D)**, and audiovisual **(B)** conditions as well as gain **(C)** controlled for the effect of age and FIQ under seven listening conditions.

Analyses of speech perception performance were carried out in the subgroups of TD children, TD adults and ASD children. We first investigated possible sex differences in speech perception performance in typically developing children of the ages five to seventeen. We submitted percent correct responses in the A and AV conditions as well as AV-gain respectively to separate repeated measures analyses of variance (RM-ANOVA) with factors SNR (5 levels of signal to noise ratio), between- subjects factor SEX (male, female) and Age (in years) as well as FIQ (full IQ) as covariates. The conditions with NN and −3 dB SNR were not included in the analysis to avoid possible ceiling effects (Ross et al., 2011). However, in order to provide the reader with an easy- to interpret characterization of the group differences, we displayed A and AV speech perception performance as well as AV-gain as it unfolded over all intelligibility conditions (Figure 1). Since the speech reading condition (V) was only presented without noise a separate univariate ANOVA was carried with Sex as a main factor and Age and FIQ serving as covariates. For all ANOVAs we assured the absence of violations of assumptions of equality of variances and equality of covariance matrices (Box test). Violations of the sphericity assumption of the RM-ANOVA were corrected by adjusting the degrees of freedom with the Greenhouse-Geisser correction method. All results relevant to our hypotheses are reported in the main text of the results section and the full statistical report can be found in the tables. We expected significant main effects of condition, SNR level, group and age as well as an interaction between condition and SNR level replicating previous findings (Ross et al., 2007a,b, 2011; Ma et al., 2009; Foxe et al., 2015). Audiovisual enhancement (or AV-gain) was operationalized here as the difference in performance between the AV and the A-alone condition (AV – A). In a second step similar analyses were carried out in the subgroup of neurotypical adults to determine if possible sex differences persist into adulthood. Information on IQ was not available and did not serve as a covariate in this test. For the assessment of sex differences in ASD children IQ information was only available for a small subset of ASD females (see Table 1) and therefore only age was included as a covariate.

## Results

### Sex differences in TD children and adults

#### Auditory alone

As reported previously (Ross et al., 2007a,b, 2011; Foxe et al., 2015) and can be seen in Figure 1, performance in the A condition showed a monotonous, close to linear increase from near zero percent correct at the lowest SNR (males: *M* = 0.71%, *SD* = 5.27%; females: *M* = 1.5%, *SD* = 2.26%) to approximately 90% correct word identification when no noise was added (males: *M* = 86.9%, *SD* = 2.45%; females: *M* = 88.2%, *SD* = 9.6%). Visual inspection revealed that females performed slightly better at intermediate SNRs. These small differences in performance, however, only approached significance [*F*_(1, 98)_ = 3.77; *p* = 0.055; η^2^_*p*_ = 0.037]. Both covariates Age [*F*_(1, 98)_ = 23.17; *p* < 0.001; η^2^_*p*_ = 0.19] and FIQ [*F*_(1, 98)_ = 7.8; *p* = 0.006; η^2^_*p*_ = 0.07] had significant main effects on performance. Age [*F*_(3, 294.66)_ = 8.68; *p* < 0.001; η^2^_*p*_ = 0.08] and FIQ [*F*_(3, 294.66)_ = 3.16; *p* = 0.025; η^2^_*p*_ = 0.03] showed significant interactions with SNR. Under the restricted range of SNR levels, SNR did not show an independent main effect [*F*_(3, 98)_ = 0.88; *p* = ns.]. We tested whether the effect of sex was also present in our sample of healthy 28 adult men and 28 adult women between the ages of 20–38 years but could not find statistical evidence for group differences [main effect Sex: *F*_(1, 53)_ = 0.001; *p* = ns.] (see Table 3). Even in the group of adults, age had a significant main effect on performance [main effect Age: *F*_(1, 53)_ = 7.43; *p* = 0.009; η^2^_*p*_ = 0.12]. Interestingly, the RM- ANOVA returned a significant interaction between SNR and Sex [*F*_(2.87, 152.33)_ = 3.55; *p* = 0.017; η^2^_*p*_ = 0.06]. However, a subsequent inspection of the age- corrected performance revealed that interactions were not uniform across SNRs.

**Table 3:** **Auditory- alone performance as a function of Sex, Age, and SNR in TD adults**.

**Source**	***SS***	***df***	***MS***	***F***	***p***	**η^2^_*p*_**
**TESTS OF BETWEEN-SUBJECTS EFFECTS**						
Age	1538.616	1	1538.616	7.514	0.008	0.124
Sex	13.14	1	13.14	0.064	0.801	0.001
Error	10852.64	53	204.767			
**TESTS OF WITHIN-SUBJECTS EFFECTS**						
SNR	4851.007	2.858	1697.483	11.424	0.000	0.177
SNR × Age	496.924	2.858	173.897	1.120	0.322	0.022
SNR × Sex	1668.784	2.858	583.947	3.930	0.011	0.069
Error	22506.15	151.462	148.593			

#### Audiovisual

Visual inspection of Figure 1B reveals that speaker articulation substantially improved speech intelligibility. Participants correctly identified approximately 20% of the words at the lowest SNR (males: *M* = 17.84%, *SD* = 10.6%; females: *M* = 22.32%, *SD* = 10.62%) and approximately 90% without noise (males: *M* = 88.71%, *SD* = 8.83%; females: *M* = 94.25%, *SD* = 8.84%). Females performed better across all SNR conditions which was confirmed by a significant main effect of Sex with substantially larger effect size than the group differences in the A condition [*F*_(1, 98)_ = 17.65; *p* < 0.001; η^2^_p_ = 0.15]. Again, factors Age [*F*_(1, 98)_ = 72.14; *p* < 0.001; η^2^_p_ = 0.42] and FIQ [*F*_(1, 98)_ = 9.79; *p* = 0.002; η^2^_p_ = 0.09] had significant main effects on performance. The parametric variation of noise produced a monotonic linear increase in performance between best and worst listening conditions which was confirmed by a significant main effect of SNR [*F*_(3.45, 338.35)_ = 3.05; *p* = 0.023; η^2^_p_ = 0.03]. The RM-ANOVA did not return interactions other than between SNR and Sex [*F*_(3.45, 338.35)_ = 2.77; *p* = 0.034; η^2^_p_ = 0.027]. For a full report, please refer to Table 4.

**Table 4:** **Audiovisual performance as a function of Sex, Age, FIQ and SNR in TD children**.

**Source**	***SS***	***df***	***MS***	***F***	***p***	**η^2^_*p*_**
**TESTS OF BETWEEN-SUBJECTS EFFECTS**						
Age	25291.845	1	25291.845	72.139	0.000	0.424
FIQ	3431.731	1	3431.731	9.788	0.002	0.091
Sex	6188.091	1	6188.091	17.650	0.000	0.153
Error	34358.639	98	350.598			
**TESTS OF WITHIN-SUBJECTS EFFECTS**						
SNR	1252.243	3.453	362.696	3.054	0.023	0.030
SNR × Age	290.076	3.453	84.017	0.707	0.567	0.007
SNR × FIQ	402.776	3.453	116.659	0.982	0.409	0.010
SNR × Sex	1134.769	3.453	328.671	2.767	0.034	0.027
Error	40184.161	338.355	118.763			

In TD adults there was no evidence for sex differences in the AV condition [*F*_(1, 53)_ = 0.23; *p* = ns.] and there was no significant effect of factor Age [*F*_(1, 53)_ = 1.24; *p* = ns.] (see Table 5 for the full report).

**Table 5:** **Audiovisual performance as a function of Sex, Age, and SNR in TD adults**.

**Source**	***SS***	***df***	***MS***	***F***	***p***	**η^2^_*p*_**
**TESTS OF BETWEEN-SUBJECTS EFFECTS**						
Age	604.772	1	604.772	1.242	0.270	0.023
Sex	111.137	1	111.137	0.228	0.635	0.004
Error	25798.587	53	486.766			
**TESTS OF WITHIN-SUBJECTS EFFECTS**						
SNR	2027.801	3.808	532.460	4.243	0.003	0.074
SNR × Age	215.482	3.808	56.581	0.451	0.762	0.008
SNR × Sex	996.300	3.808	261.609	2.085	0.087	0.038
Error	25328.390	201.843	125.486			

#### Audiovisual gain

Conforming with previous reports (Ross et al., 2007a,b, 2011; Foxe et al., 2015), audiovisual gain showed an inverted u-shaped curvilinear relationship with a maximum at intermediate SNRs at −9 dB in male and −12 dB in female participants (see Figure 1C). While substantial AV-gain was achieved at the lowest SNR (17% in males, 21% in females), AV-gain decreased as AV-performance approached ceiling. While AV-gain was very similar in male and female participants at SNRs above −12 dB, it was larger in females at the three lowest SNRs which was reflected in a significant main effect of factor Sex on AV-gain [*F*_(1, 98)_ = 5.39; *p* = 0.022; η^2^_p_ = 0.05]. Factor Age had a significant main effect on performance [*F*_(1, 98)_ = 17.49; *p* < 0.001; η^2^_p_ = 0.15] whereas FIQ did not [*F*_(1, 98)_ = 0.91; *p* = ns.]. The RM-ANOVA also returned a significant interaction between factors Age and SNR [*F*_(3.32, 325.32)_ = 3.81; *p* = 0.008; η^2^_p_ = 0.037]. Please refer to Table 6 for a full report. We found no evidence for differences between males and females in our adult sample [*F*_(1, 53)_ = 0.11; *p* = ns.] (Table 7).

**Table 6:** **Audiovisual gain (AV-A) as a function of Sex, Age, FIQ, and SNR in TD children**.

**Source**	***SS***	***df***	***MS***	***F***	***p***	**η^2^_*p*_**
**TESTS OF BETWEEN-SUBJECTS EFFECTS**						
Age	9140.233	1	9140.233	17.486	0.000	0.151
FIQ	474.202	1	474.202	0.907	0.343	0.009
Sex	2818.225	1	2818.225	5.392	0.022	0.052
Error	51225.099	98	522.705			
**TESTS OF WITHIN-SUBJECTS EFFECTS**						
SNR	1324.159	3.320	398.897	1.945	0.116	0.019
SNR × Age	2595.396	3.320	781.851	3.811	0.008	0.037
SNR × FIQ	428.268	3.320	129.014	0.629	0.613	0.006
SNR × Sex	1607.347	3.320	484.206	2.360	0.065	0.024
Error	66734.181	325.316	205.136			

**Table 7:** **Audiovisual gain (AV-A) as a function of Sex, Age, and SNR in TD adults**.

**Source**	***SS***	***df***	***MS***	***F***	***p***	**η^2^_*p*_**
**TESTS OF BETWEEN-SUBJECTS EFFECTS**						
Age	214.127	1	214.127	0.494	0.485	0.009
Sex	47.848	1	47.848	0.110	0.741	0.002
Error	22985.405	53	433.687			
**TESTS OF WITHIN-SUBJECTS EFFECTS**						
SNR	864.143	3.236	267.022	0.801	0.503	0.015
SNR × Age	921.229	3.236	284.662	0.854	0.473	0.016
SNR × Sex	1369.585	3.236	423.205	1.270	0.286	0.023
Error	57149.268	171.520	333.194			

#### Speechreading

Females (*M* = 13.79%, *SD* = 7.82) performed significantly better than males (*M* = 8.29%, *SD* = 7.79) under conditions where only visual articulation was provided and when performance was adjusted for the effect of age and FIQ [*F*_(1, 98)_ = 8.59; *p* = 0.001; η^2^_p_ = 0.11] (see Figure 1D). The effect of age was strong [*F*_(1, 98)_ = 18.86; *p* < 0.001; η^2^_p_ = 0.16], but the main effect of factor FIQ did not reach significance [*F*_(1, 98)_ = 1.95; *p* = ns.]. Much to our surprise, we found that male TD adults (*M* = 13.09%; *SD* = 8.1) performed better in the speechreading condition than TD females (*M* = 8.14%; *SD* = 8.1) [*F*_(1, 56)_ = 6.8; *p* = 0.027].

### Performance differences between ASD males and females

Included in this analysis were 58 ASD males and 15 ASD females between the ages of 8 and 15 years of age. The statistical analysis was equivalent to the on in the report on TD children above. Important to note here is that on the basis of the direction of the sex effect in our TD sample and our relatively small subsample of ASD females statistical tests were carried out one-sided.

#### Auditory alone

**Figure 2:**
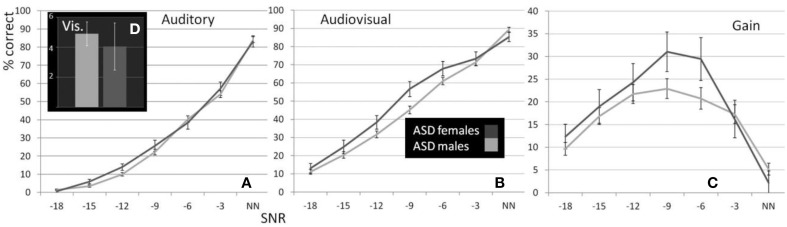
**Performance of male and female ASD children**. Estimated Marginal Means (at Age 11.01) for ASD males and females (ages 8–15) for auditory **(A)**, visual **(D)**, and audiovisual **(B)** conditions as well as gain **(C)** controlled for the effect of age under seven listening conditions.

The graph depicting A performance in male and female participants diagnosed with ASD (Figure 2A) shows that both groups performed very similarly in this condition. This was confirmed by the RM- ANOVA that returned no significant main effect of factor Sex [*F*_(1, 70)_ = 0.99; *p* = ns.]. Besides a significant effects of factor Age [*F*_(1, 70)_ = 7; *p* = 0.01, η^2^_p_ = 0.09] and an interaction with SNR [*F*_(2.59, 181.08)_ = 5.9; *p* = 0.001, η^2^_p_ = 0.08] no other effect was observed. A full RM-ANOVA report can be found in Table 8.

**Table 8:** **Auditory- alone performance as a function of Sex, Age and SNR in ASD children (8–15 years)**.

**Source**	***SS***	***df***	***MS***	***F***	***p***	**η^2^_*p*_**
**TESTS OF BETWEEN-SUBJECTS EFFECTS**						
Age	846.352	1	846.352	6.998	0.005	0.091
Sex	119.245	1	119.245	0.986	0.162	0.014
Error	8465.783	70	120.940			
**TESTS OF WITHIN-SUBJECTS EFFECTS**						
SNR	426.352	2.587	164.812	1.487	0.001	0.021
SNR × Age	1692.133	2.587	654.118	5.903	0.000	0.078
SNR × Sex	306.663	2.587	118.545	1.070	0.178	0.015
Error	20066.097	181.083	110.812			

#### Audiovisual

In this test, the assumption of homogeneity of variances was violated at the lowest SNR. We therefore conducted a test including performance at −15, −12, −9, −6, and −3 dB SNRs. Unlike the performance in the auditory alone condition, sex differences were evident in the presence of visual speech which was consistent at all SNRs except without noise, when performance was near ceiling levels (see Figure 2B). The group difference was confirmed by a significant main effect of Sex in the RM- ANOVA [*F*_(1, 70)_ = 3.48; *p* = 0.033; η^2^_p_ = 0.047]. Besides significant effects of factors Age [*F*_(1, 70)_ = 19.96; *p* < 0.001; η^2^_p_ = 0.22] and SNR [*F*_(3.8, 265.38)_ = 12.34; *p* < 0.001; η^2^_p_ = 0.15] no other sources reached our significance criterion. The complete RM-ANOVA report can be found in Table 9.

**Table 9:** **Audiovisual performance as a function of Sex, Age, and SNR in ASD children (8–15 years)**.

**Source**	***SS***	***df***	***MS***	***F***	***p***	**η^2^_*p*_**
**TESTS OF BETWEEN-SUBJECTS EFFECTS**						
Age	13032.844	1	13032.844	19.962	0.000	0.222
Sex	2274.873	1	2274.873	3.48	0.033	0.047
Error	45702	70	652.892			
**TESTS OF WITHIN-SUBJECTS EFFECTS**						
SNR	5367.008	3.711	1446.307	12.346	0.000	0.15
SNR × Age	182.53	3.711	49.188	0.42	0.78	0.006
SNR × Sex	570.17	3.711	153.65	1.312	0.266	0.018
Error	30430.788	259.759	117.15			

#### Audiovisual gain

The overall difference between males and females with ASD was also apparent in the graph depicting audiovisual gain for both groups across SNRs (Figure 2C). The overall difference between males and females was significant [*F*_(1, 70)_ = 2.91; *p* = 0.047; η^2^_p_ = 0.04]. Factors Age [*F*_(1, 70)_ = 14.06; *p* < 0.001; η^2^_p_ = 0.17] and SNR [*F*_(3.35, 234.54)_ = 4.15; *p* = 0.003; η^2^_p_ = 0.06] and the interaction between Age and SNR [*F*_(3.35, 234.54)_ = 3.29; *p* = 0.009; η^2^_p_ = 0.045] were also observed to explain a significant amount of variance in audiovisual gain. A full RM-ANOVA report can be found in Table 10.

**Table 10:** **Audiovisual gain (AV-A) as a function of Sex, Age, and SNR in ASD children (8–15 years)**.

**Source**	***SS***	***df***	***MS***	***F***	***p***	**η^2^_*p*_**
**TESTS OF BETWEEN-SUBJECTS EFFECTS**						
Age	6532.272	1	6532.272	14.062	0.000	0.167
Sex	1349.714	1	1349.714	2.906	0.047	0.040
Error	32516.205	70	464.517			
**TESTS OF WITHIN-SUBJECTS EFFECTS**						
SNR	2876.234	3.351	858.446	4.154	0.003	0.056
SNR × Age	2276.766	3.351	679.528	3.288	0.009	0.045
SNR × Sex	490.775	3.351	146.478	0.709	0.283	0.010
Error	48465.726	234.536	206.645			

#### Speechreading

In the speechreading condition male participants with ASD (*M* = 4.9%, *SD* = 6.01) performed slightly (*M* = 4.05%, *SD* = 6.08) but not significantly better than females [*F*_(1, 70)_ = 0.23; *p* = ns.]. The bar graph in Figure 2D depicts performances in speechreading corrected for age.

#### Eye-movement analyses

**Figure 3:**
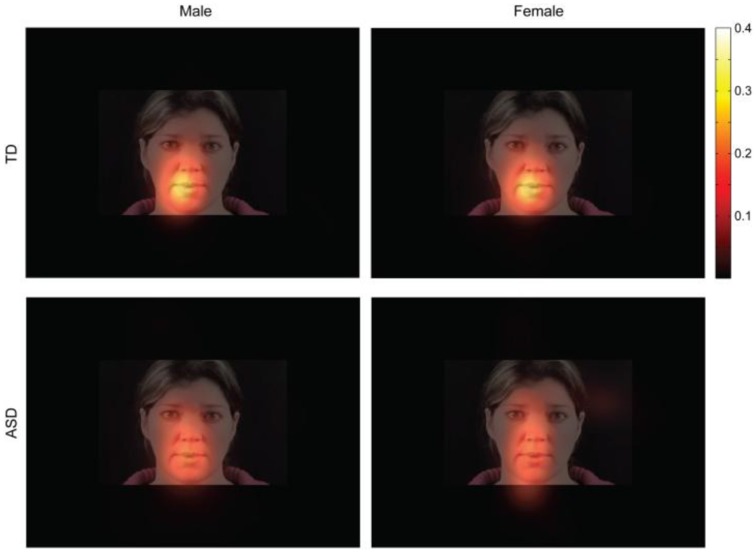
**Fixation maps for fixations during presentation of the speech stimuli**. Data for participants from all age ranges were combined. Brighter colors indicate a higher consistency of fixations. The theoretical maximum value is 1. This value can only be reached if all participants fixate on exactly the same spot during all trials and during each fixation.

There were no differences in viewing behavior between female and male participants in the TD (*t*_(85)_ = −0.569, *p* = ns.) and ASD (*t*_(60)_ = −0.069, *p* = ns.) groups. Proportions of fixations (Figure 3) on different parts of the face as well as number of fixations were not statistically distinguishable between sexes (Table 11). We also found no differences in the mean fixation times between TD males (734 ms) and TD females (678 ms) *t*_(85)_ = 0.54, *p* = ns. and between ASD males (586 ms) and ASD females (549 ms) *t*_(85)_ = 0.44, *p* = ns.

**Table 11:** **Comparison of Eye-Tracking between males and females by diagnostic group**.

	**TD males**	**TD females**	**ASD males**	**ASD females**
n	49	38	55	7
Number fixations	2.2	2.3 (*p* > 0.05)	2.4	2.5 (*p* > 0.05)
% Fixations face	93.0	87.7 (*z* < 0.9)	81.8	79.1 (*z* < 0.2)
% Fixations eyes	12.6	9.5 (*z* < 0.5)	12.9	10.1 (*z* < 0.3)
% Fixations mouth	46.0	49.5 (*z* < 0.4)	35.9	25.8 (*z* < 0.6)

Therefore, it is very unlikely that the behavioral results can be explained by one group attending the stimuli less.

### Comparison of performances of non-adult subgroups

**Figure 4:**
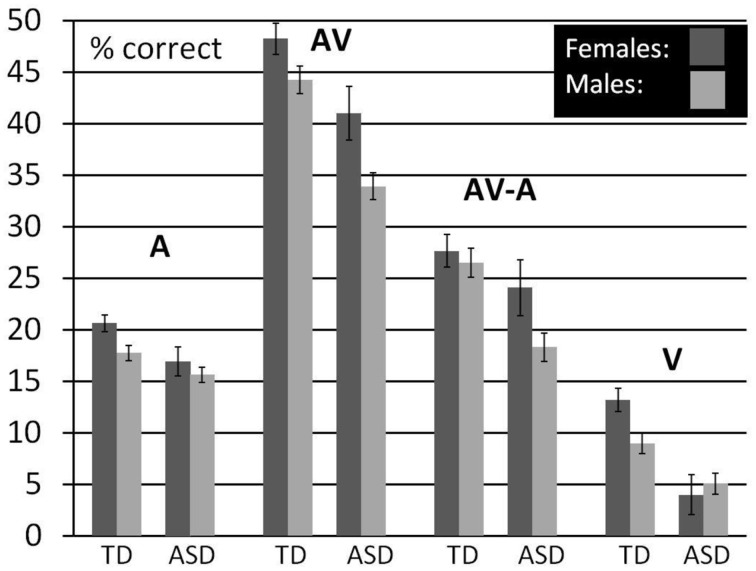
**Comparison between males and females by diagnostic group**. Estimated Marginal Means (at Age 11.02) for TD and ASD males and females (ages 8–15) for A, V, and AV conditions as well as AV-A controlled for the effect of age.

With this descriptive analysis we aim to provide the opportunity for a more direct comparison of performance differences between males and females and the association with diagnostic status. For this, we averaged performance in each condition over the lower five SNRs with the exception of V- performance which was presented only without noise. We restricted the age- range to 8–15 years but also included children for which we had no IQ scores. We did not control for FIQ in this analysis because we did not obtain IQ scores for all the females in our ASD sample. We used a General Linear Model to establish estimated performance scores that were corrected for age. Figure 4 (see also Table 12) shows a bar graph depicting mean performance for A, AV, and V conditions as well as ^****^AV-gain for *n* = 98 typically developing children (*n* = 43 females, *n* = 55 males) and *n* = 73 children diagnosed with ASD (*n* = 15 females; *n* = 58 males).

**Table 12:** **Comparison between males and females by diagnostic group**.

	**TD males**	**TD females**	**ASD males**	**ASD females**
n	55	43	58	15
A	17.97 (0.81)	20.45 (0.93)	15.33(0.7)	17.53 (1.11)
V	9.22 (1.21)	13.02 (1.28)	4.76 (0.83)	4.61 (1.38)
AV	44.81 (1.4)	47.86 (1.39)	33.28 (1.51)	42.43 (3.74)
AV-A	26.84 (1.52)	27.42(1.58)	17.94 (1.31)	24.9 (3)

*Cell values represent mean performances and standard error of the mean (SE) at the lower 5 SNRs for A, V, AV conditions and AV-A*.

Visual inspection of the bar graphs reveals that, conforming with our findings reported earlier, both ASD diagnosis and gender were consistently associated with performance with overall lower performance in association with ASD diagnosis and male sex (with the exception of male performance in the V-condition). These associations were most pronounced in the AV condition. The graphs also suggest that the association between ASD diagnosis and lower performance was more pronounced in males than in females. In other words, the difference between TD males and ASD males was larger than the difference between TD females and ASD females in all conditions except speechreading. While ASD females still performed consistently lower than TD males, differences to TD males appeared small. For a statistical verification of these small differences a larger sample size for ASD females is warranted.

## Discussion

In this study, we assessed whether male and female participants differed in their ability to benefit from visual speech when target words were presented under varying background noise conditions. We tested sex differences in a sample of TD children and adults, and children diagnosed with ASD. In the TD child sample, females were significantly superior in recognizing speech in noise under auditory alone conditions than were males. Even larger performance differences were found under multisensory conditions, with the females benefitting significantly more from the addition of visual speech than the males, particularly under low intelligibility conditions (i.e., higher background noise). The females also performed better under pure speechreading conditions. These sex differences in children were fully absent in the sample of adult participants with the exception of the speechreading condition, in which case the males were slightly but significantly better at speechreading than the females. We then tested whether male/female performance differences were present in a sample of ASD children and found that ASD females performed significantly better under audiovisual conditions than ASD males, a difference that was not apparent for the auditory-alone condition in which no visual articulatory information was provided. Similarly, we found no evidence for sex differences in the ASD sample in speechreading, thus ruling out a purely unisensory account of differences in multisensory gain. Further, eye-tracking data made it clear that these sex differences were not due to different gaze patterns.

Clearly, multisensory speech perception is an important aspect of social communication. Therefore, possible answers to the observed sex differences might be found in sex differences in the development of social communication skills in general. Indeed, there is an extensive literature on the development of social communication in males and females which most frequently shows that females display greater, or at least earlier, development of skills in this domain. On average, females start to talk earlier than males (Fenson et al., 1994) and score higher on tests of verbal fluency (Hyde and Linn, 1988). Girls and women exhibit more eye contact than males (Hall, 1985), show greater ability to detect and understand emotional facial expressions (Rosenthal et al., 1979; Happe, 1995; Baron-Cohen et al., 1997, 1999) and there is accumulating evidence that preadolescent girls show relatively higher abilities in tasks assessing social understanding such as inferring other people's mental states (Theory of Mind; Hatcher et al., 1990; Bosacki and Astington, 1999; Calero et al., 2013).

It has been suggested that differences in social communication may have their origins at the earliest stages of development during intrauterine exposure to sex hormones (Auyeung et al., 2006, 2009; Chapman et al., 2006) thereby affecting brain structure and function relevant to social communication. Female newborns look longer at animated faces than mobile mechanical objects whereas newborn males showed the opposite pattern (Connellan et al., 2000). These genetic/epigenetic/hormonal origins of sex differences may be further enhanced by differential socialization, especially by parents (Stern and Karraker, 1989). Mothers have more verbal communication with their daughters than with their sons (Leaper et al., 1998) and parents show preferential acknowledgement of their infant daughter's emotional displays than their son's (Malatesta and Haviland, 1982). These factors may explain why female toddlers and infants show greater nonverbal communication skills (Clarke-Stewart, 1973; Fenson et al., 1994), vocabulary acquisition (Huttenlocher et al., 1991) and frequency of social initiations (Klein and Durfee, 1978). The evidence for differences in integration abilities between males and females remains far from conclusive, although women have been shown to be better at lip reading when target words were presented in a sentence context (Johnson et al., 1988; Watson et al., 1996). Similarly, some investigators have reported increased sensitivity to the so-called McGurk effect in women (Aloufy et al., 1996; Öhrström and Traunmüller, 2004). The McGurk illusion refers to a rather dramatic multisensory perceptual phenomenon whereby presentation of incongruent visual articulatory inputs can greatly modify the speech sound that is heard (McGurk and MacDonald, 1976; Saint-Amour et al., 2007). Irwin et al. (2006) showed that women displayed greater influence of visual speech on heard speech than men, but did not find evidence for sex differences in speechreading abilities.

Apart from greater abilities in AV-speech perception in ASD girls than ASD boys, our findings suggest that AV-benefit is not as affected by ASD in females as in males when stratifying performance by sex. Small relative decrements in AV-benefit in ASD females compared to TD males may exist, but did not reach statistical significance. ASD females do show lower performance than their unaffected counterparts, but they appear to be affected to a lesser extent than their male peers. Given our evidence for a sexual dimorphism in audiovisual speech perception in the TD population, and the apparent role of audiovisual speech perception for social communication (e.g., reading speech cues from faces) one may be inclined to interpret these findings within the framework of the EMB- theory. However, a closer look reveals that our pattern of results does not entirely conform to the EMB- theory. Although, to our knowledge, this prediction has not been explicitly stated by EMB proponents, it is implicit in the theory that if ASD brings about an increase or exaggeration of a masculine phenotype then one would expect that the effect of the disorder should have a greater impact on females than on males since males with ASD approach the extreme end of the proposed “maleness” spectrum and the difference between affected and unaffected males might therefore be subject to a ceiling effect. In contrast to this prediction, our data show larger differences in males than in females. However, the interpretation of our findings in light of the EMB theory should be exercised with caution, since it is not entirely clear whether the ability to read visual speech cues can be interpreted as an act of “empathizing” as has been proposed for the ability to read emotional expressions on people's faces (Baron-Cohen et al., 2005).

The pattern of our findings suggests a more parsimonious model of the observed sex differences. AV-speech perception can be regarded as an instance of language and social communication for which, as discussed above, previous evidence for a sexual phenotypic difference has been provided. The ability to integrate visual speech might therefore have genetic as well as environmental origins such as differential parental socialization resulting in an earlier development of these abilities in females and a sustained advantage into the late teenage years (Ross et al., 2011) relative to age-matched males. This developmental trajectory flattens toward early adulthood when females reach their performance maximum which allows males to “catch up” explaining the absence of performance differences in adults. ASD imposes a developmental delay in both males and females that resolves in the early teenage years rather than an irreversible and sustained impairment (Foxe et al., 2015). The fact that females with ASD are affected to a lesser extent might be afforded by “protective factors” inherent in the female genotype (Skuse et al., 1997; Skuse, 2000; Robinson et al., 2013). Unfortunately, the exploration of the developmental course of AV speech processing stratified by sex was not possible within the framework of this study due to the low subject numbers in our female ASD sample and should be subject to future studies.

High-density recordings of electrophysiological brain activity have revealed that the neural integration of multisensory inputs is impaired in children with ASD (Russo et al., 2010; Brandwein et al., 2013, 2015), and it is also the case that ASD children are not as effective at deploying attention to a relevant unisensory stream when there are competing multisensory inputs (Murphy et al., 2014). These studies included only a small proportion of female participants with ASD, precluding consideration of the role of sex in these deficits. An important question then is whether these deficits are equivalently seen in both male and female participants with ASD, or if they are sexually dimorphic as we see in the present study. These electrophysiological studies presented simple stimuli of no obvious higher-order communicative or social significance, and as such the findings were interpreted as representing the breakdown of basic sensory and attentional processes, although these could well have cascading consequences for higher-order functions such as multisensory speech perception. It is instructive that even fundamental deficits in multisensory integration processes and in the basic sensory processing of auditory tonal stimuli were found to be related to the severity of clinical symptoms in ASD children (Brandwein et al., 2015). This would suggest to us the possibility that while impaired communication among sensory cortices is part of the broader autism phenotype, protective factors may serve to “rescue” multisensory speech processing functions in females with ASD. Alternatively, it is also possible that even basic multisensory integrative processing is spared in females with ASD. Future work will be required to determine the extent to which this sparing is observed for other types of multisensory integrative processes, and whether it extends to non-social processing.

A limitation of this study is that the generalization of our findings from our ASD sample is only possible to the population of high functioning individuals with ASD. Using tasks adapted to individuals with low functioning ASD, future research may determine whether sex differences can also be observed in this population.

In conclusion this study provides evidence for sex differences in the ability to integrate heard and seen speech under noisy environmental conditions in a large sample of typically developing children and teenagers between the ages of 5 and 17 years. These differences were absent in a sample of healthy adults. We further show that multisensory speech processing is less affected in ASD females than males. Our findings underline the importance of considering sex differences in the search for autism endophenotypes. An advanced understanding of sex differences in ASD may benefit our understanding of the genetic, neurobiological and environmental factors involved in the development of ASD. Basic research on sex differences in ASD has practical implications for therapeutic intervention and may inform clinicians to delineate more personalized treatments for this diverse disorder.

## Author contributions

JF, LR, and SM conceived the project, analyzed the data and wrote the paper. VDB and LR collected the data. VDB provided support with editing the manuscript. LR constructed the stimulus set, and HPF conducted and analyzed the eye-tracking component of the experiments. All authors discussed the results, commented on the manuscript and approved the final version.

### Conflict of interest statement

The authors declare that the research was conducted in the absence of any commercial or financial relationships that could be construed as a potential conflict of interest.
